# A Novel *Pseudomonas geniculata* AGE Family Epimerase/Isomerase and Its Application in d-Mannose Synthesis

**DOI:** 10.3390/foods9121809

**Published:** 2020-12-06

**Authors:** Zhanzhi Liu, Ying Li, Jing Wu, Sheng Chen

**Affiliations:** 1 State Key Laboratory of Food Science and Technology, Jiangnan University, 1800 Lihu Avenue, Wuxi 214122, China; liu.z@jiangnan.edu.cn (Z.L.); yingli202012@163.com (Y.L.); jingwu@jiangnan.edu.cn (J.W.); 2 School of Biotechnology and Key Laboratory of Industrial Biotechnology Ministry of Education, Jiangnan University, 1800 Lihu Avenue, Wuxi 214122, China

**Keywords:** AGE family epimerase/isomerase, d-mannose isomerase, Identification, d-mannose

## Abstract

d-mannose has exhibited excellent physiological properties in the food, pharmaceutical, and feed industries. Therefore, emerging attention has been applied to enzymatic production of d-mannose due to its advantage over chemical synthesis. The gene *age* of *N*-acetyl-d-glucosamine 2-epimerase family epimerase/isomerase (AGEase) derived from *Pseudomonas geniculata* was amplified, and the recombinant *P. geniculata* AGEase was characterized. The optimal temperature and pH of *P. geniculata* AGEase were 60 °C and 7.5, respectively. The *K*_m_, *k*_cat_, and *k*_cat_/*K*_m_ of *P. geniculata* AGEase for d-mannose were 49.2 ± 8.5 mM, 476.3 ± 4.0 s^−1^, and 9.7 ± 0.5 s^−1^·mM^−1^, respectively. The recombinant *P. geniculata* AGEase was classified into the YihS enzyme subfamily in the AGE enzyme family by analyzing its substrate specificity and active center of the three-dimensional (3D) structure. Further studies on the kinetics of different substrates showed that the *P. geniculata* AGEase belongs to the d-mannose isomerase of the YihS enzyme. The *P. geniculata* AGEase catalyzed the synthesis of d-mannose with d-fructose as a substrate, and the conversion rate was as high as 39.3% with the d-mannose yield of 78.6 g·L^−1^ under optimal reaction conditions of 200 g·L^−1^ d-fructose and 2.5 U·mL^−1^ *P. geniculata* AGEase. This novel *P. geniculata* AGEase has potential applications in the industrial production of d-mannose.

## 1. Introduction

d-mannose is an epimer of d-glucose with the isomerization at the C-2 position [1]. It is in a free state in various fruit peels including orange, peach, and apple. It does not cause significant increases in blood sugar because it cannot be well metabolized in the body, and it has been extensively employed in the food, pharmaceutical, and feed industries [2,3]. Recently, researchers described the application of d-mannose as a good alternative in the prevention and treatment of urinary tract infections (UTIs) [4,5]. d-mannose can be extracted from jujube, apple pulp, and orange peel [6,7,8]. However, this method is complicated and generates low yield (jujube 12.9% [6], apple 2.3–4.0% [7], and orange peel 6.1% [8]) and high production costs. Through chemical methods, d-mannose can be produced from d-glucose in 1% molybdate under acidic conditions. The conversion rate of this method is about 32.0% [9]. However, this method produces a large amount of acidic waste liquid that pollutes the environment, and the final separation and purification is also challenging.

Enzymatic synthesis is an alternative method that uses d-fructose or d-glucose as the substrate to produce d-mannose via enzyme catalysis. This method has advantages such as mild reaction conditions, fewer byproducts, easy separation and purification processes, no environmental pollutants, and low cost, and plays an increasingly important role in the preparation of d-mannose [10]. The *N*-acetyl-d-glucosamine 2-epimerase family epimerase/isomerase (AGEase) includes *N*-acetyl-d-glucosamine 2-epimerase, Cellobiose 2-epimerase, and YihS enzyme subfamilies that all catalyze isomerization at the C-2 position [11,12,13]. The catalytic substrate of *N*-acetyl-d-glucosamine 2-epimerase is the modified sugar *N*-acetyl-d-mannosamine, which can be isomerized into *N*-acetyl-d-glucosamine [11]. Cellobiose 2-epimerase can catalyze oligosaccharides containing β-1,4 glycoside bonds, such as cellobiose, lactose, 4-O-beta-d-mannose-d-glucose, etc. [12]. YihS enzyme exhibits aldose-ketose isomerase activity, catalyzing isomerization of unmodified oligosaccharides such as d-mannose, d-fructose, and d-glucose, as well as threose and xylulose [13]. Several enzymes have been reported to be useful for producing d-mannose, including d-mannose isomerase (d-MIase, EC 5.3.1.7), d-lyxose isomerase (d-LIase, EC 5.3.1.15), and cellobiose 2-epimerase (CEase, EC 5.1.3.11), which belongs to AGEase and produces d-mannose with d-fructose or d-glucose as substrate (Table 1) [14,15,16,17,18]. Identification of novel d-mannose producing enzymes is essential for the enhancement of d-mannose production.

In this study, a gene *age* encoding for AGEase was cloned from *Pseudomonas geniculata*. The recombinant *P. geniculata* AGEase was investigated and identified as d-MIase. The property of *P. geniculata* AGEase for producing d-mannose was also characterized.

## 2. Materials and Methods

### 2.1. Strains, Chemicals, and Enzymes

*P. geniculata* containing the AGEase gene was stored in our laboratory. The cloning host *Escherichia coli* JM109 and the expression host *E. coli* BL21 (DE3) were purchased from Bao Bioengineering Co., Ltd. (Dalian, China). The vector pET-24a was purchased from Novagen Co., Ltd. (Shanghai, China). The Plasmid pET-24a-*age* was constructed by this study. The restriction enzymes (*Nde* I and *Hind* III), DNA polymerase Primer STAR^®^ HS, T4 DNA ligase, rTaq enzyme, and alkaline phosphatase CIAP were purchased from Bao Bioengineering Co., Ltd. (Dalian, China). The DNA recovery kits, PCR product purification kits, and Plasmid extraction kits were purchased from Tiangen Biochemical Technology Co., Ltd. (Shanghai, China). All chemicals and reagents with analytical grade were purchased from Sinopharm Chemical Reagent Co., Ltd. (Shanghai, China).

### 2.2. Construction of Recombinant Plasmid

Two oligonucleotide primers (F: 5′-TATACATATGAGCACCTCGCCCGATTTC-3′, R: 5′-GCCGCAAGCTTCAACGCACCACGTTCAAC-3′), with restriction sites *Nde* I and *Hind* III, were utilized to amplify the gene *age* by PCR using the whole *P. geniculata* genome as a template. After purification and digestion, the PCR product was digested and ligated into the vector pET-24a. The recombinant plasmid pET-24a-*age* was transformed into *E*. *coli* BL21 (DE3) for further expression.

### 2.3. Expression of Recombinant P. geniculata AGEase

A single colony of recombinant *E. coli* BL21 (DE3) containing pET-24a-*age* plasmid was inoculated in 10 mL LB medium, and seed culture medium was obtained after being cultured for 8 h at 37 °C and 200 r·min^−1^. The seed culture was inoculated to 50 mL TB fermentation medium at a vaccination rate of 5%. The recombinant *E. coli* BL21 (DE3)/pET-24a-*age* was cultured at 37 °C for 1.5–2 h until the OD_600_ reached 0.6. Inducer Isopropyl β-d-Thiogalactoside (IPTG) at a 0.1 mM final concentration was added, then the culture was induced at 25 °C for 24 h. All of the cultures mentioned above contained 100 μg·mL^−1^ Kanamycin. The expression of *P. geniculata* AGEase was checked by 12% SDS-PAGE.

### 2.4. Purification of Recombinant P. geniculata AGEase

After flask fermentation for 24 h, 400 mL fermentation culture was centrifuged at 7741 g for 20 min. The cell pellet was resuspended in 200 mL sodium phosphate buffer (50 mM, pH 7.5). Crude enzyme solution was obtained by crushing harvested cells with a high-pressure homogenate crusher. Recombinant *P. geniculata* AGEase was precipitated by addition of 60% (NH_4_)_2_SO_4_, isolated, and purified using the AKTA protein purification system with the Superdex 200 column (column size: 10 mm × 30 cm, flow rate: 0.5 mL·min^−1^). Enzyme purity was checked by 12% SDS-PAGE. Bradford assay was used for the determination of protein concentration. The disodium hydrogen phosphate-sodium dihydrogen phosphate buffer (20 mM, pH 7.5) was used for purification. All the above solutions needed to be filtered before they could be used. All purification steps were conducted at 4 °C.

### 2.5. Recombinant P. geniculata AGEase Activity Assay

*P. geniculata* AGEase activity was determined at 60 °C by the cysteine-carbazole method [19]. A total of 900 μL 0.1 M d-mannose solution in 50 mM sodium phosphate buffer (pH 7.5) was mixed with 0.1 mL enzyme, and the reaction was performed at 60 °C for 1 min, then was terminated by adding 5 mL perchloric acid. Then, 1 mL of the above solution was mixed with 6 mL 75% (*v*/*v*) H_2_SO_4_ solution and 0.2 mL 1.5% (*w*/*v*) cysteine hydrochloride solution. Finally, 0.2 mL 0.12% carbazole ethanol solution was added and the final solution was incubated at 60 °C for 10 min, then cooled in ice water for 5 min. The absorbance at 560 nm was measured, and the concentration of d-fructose was calculated according to the d-fructose standard curve. One unit of *P. geniculata* AGEase activity is defined as the amount of enzyme required to convert d-mannose to 1 μmol of d-fructose per minute with 0.1 M of d-mannose as substrate.

### 2.6. Characterization of Purified P. geniculata AGEase

The optimal pH of *P. geniculata* AGEase was determined by measuring the activity of recombinant *P. geniculata* AGEase at different pH values using sodium phosphate buffers with different pH values (50 mM, pH 5.0–9.0), and the highest enzyme activity was defined as 100%. The *P. geniculata* AGEase was placed in sodium phosphate buffer with different pH values (50 mM, pH 5.0–10.0) and stored at 4 °C for 24 h; the enzyme activity of recombinant *P. geniculata* AGEase was measured to determine the pH stability of *P. geniculata* AGEase, and the highest activity was defined as 100%. The optimal temperature was determined by assaying the activity of *P. geniculata* AGEase at 30–70 °C, and the highest enzyme activity was defined as 100%. The *P. geniculata* AGEase was incubated at 50 °C, and samples were taken periodically to determine the residual activity of the *P. geniculata* AGEase to determine its stability at 50 °C, and the initial enzyme activity was defined as 100%. d-glucose, d-fructose, d-mannose, d-xylose, d-xylose, *N*-acetyl-d-glucosamine, and *N*-acetyl-d-mannosamine were used as substrates to determine the substrate specificity of *P. geniculata* AGEase.

### 2.7. Structure Modeling

The theoretical structure of *P. geniculata* AGEase was obtained by homology modeling utilizing the Swiss Model Protein Modeling Server [20]. These homology models were constructed by employing the crystal structure of *Marinomonas mediterranea* d-mannose isomerase (PDB ID: 5 × 32) with 57.6% similarity as the template. PyMol was utilized to visualize the generated model structures [21].

### 2.8. Determination of the Kinetic Parameters

Activity assays were conducted for different substrates at 10, 20, 30, 50, 100, 150, 200, 250, 300, 400, 500, 700, and 1000 mM. *K*_m_ (mM) and *k*_cat_ (s^−1^) of purified *P. geniculata* AGEase for substrates were calculated from Michaelis–Menten equation after fitting non-linear regression curves. Catalytic efficiency (*k*_cat_/*K*_m_) of *P. geniculata* AGEase was calculated.

### 2.9. Production of d-Mannose from d-Fructose by P. geniculata AGEase

Synthesis of d-mannose was carried out in flasks at 150 r·min^−1^ in a water bath shaker in 10 mL volume. In order to investigate the optimal temperature of d-mannose production, reactions were carried out at 40–60 °C in sodium phosphate buffer (50 mM, pH 7.5) containing 20% (*w*/*v*) d-fructose and 2.5 U·mL^−1^ *P. geniculata* AGEase. To investigate the optimal pH of d-mannose production, reactions were carried out at the optimal temperature over the pH range of 6.0–9.0 (disodium hydrogen phosphate-potassium dihydrogen phosphate buffer, 50 mM) in mixtures containing 20% (*w*/*v*) (1.1 M) d-fructose and 2.5 U·mL^−1^ (0.18 μM) *P. geniculata* AGEase for 2 h. Different amounts of *P. geniculata* AGEase were added under the same conditions of temperature, pH, substrate concentration, and time to investigate the effect of enzyme dosage on the production of d-mannose. The concentration of d-fructose was varied to investigate the optimal substrate concentration on d-mannose production under the same conditions of 50 °C, pH 7.5, and 2.5 U·mL^−1^ *P. geniculata* AGEase. 

### 2.10. High-Performance Liquid Chromatography (HPLC) Analysis of Products

An HPLC (Agilent Technologies, Santa Clara, CA, USA, NH_2_ column, 250 × 4.6 mm; Cosmosil, Japan; G1362A RID detector) was used to determine the quantities of products. After reaction, the samples were boiled for 10 min then centrifuged at 7741 g for 10 min to obtain the supernatant to measure the yield of d-mannose. All samples were filtered through 0.22 μm cellulose acetate membranes and analyzed by HPLC. The mobile phase was 75% (*v*/*v*) acetonitrile with 0.8 mL·min^−^^1^ flow rate. The yield of d-mannose in the enzyme conversion product was calculated by the external standard method.

## 3. Results and Discussion

### 3.1. Cloning and Expression of P. geniculata AGEase

**Figure 1:**
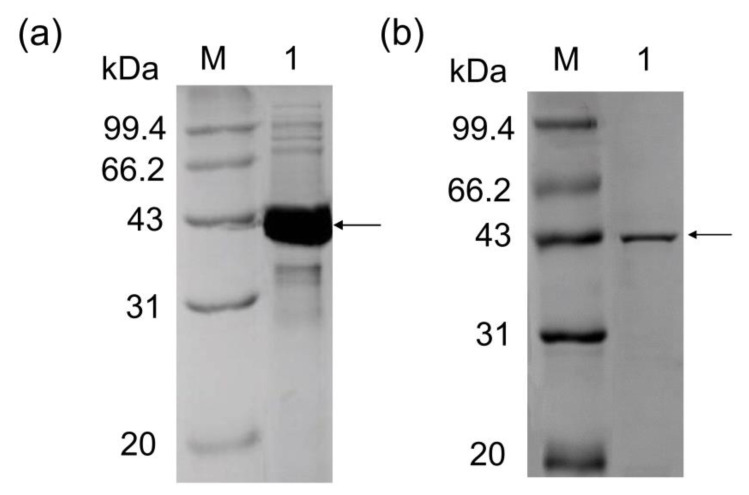
SDS-PAGE analysis of recombinant *P. geniculata* AGEase. (**a**) Lanes: M, molecular mass markers (97.4, 66.2, 43, 31, and 20 kDa). 1, lysates of whole-cell expressing recombinant *P. geniculata* AGEase after IPTG induction for 24 h. (**b**) Lanes: M, molecular mass markers (97.4, 66.2, 43, 31, and 20 kDa). 1, *P. geniculata* AGEase purified by gel column Superdex 200.

The gene *age* was cloned and *P. geniculata* AGEase was expressed in *E. coli* BL21 (DE3). The crude enzyme solutions of *P. geniculata* AGEase were obtained through cell disruption, and exhibited a 43 kDa protein band on SDS-PAGE (Figure 1a). This result demonstrated the successful expression of *P. geniculata* AGEase in *E. coli* BL21 (DE3).

### 3.2. Purification of P. geniculata AGEase and Identification of Biochemical Properties

**Figure 2:**
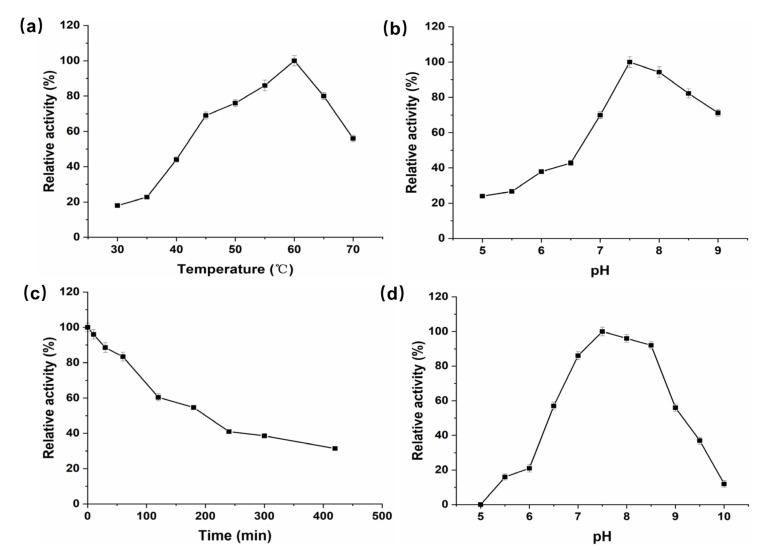
Enzymatic properties of recombinant *P. geniculata* AGEase. Optimal temperature for *P. geniculata* AGEase (**a**), the reactions were performed in 50 mM sodium phosphate buffer (pH 7.5) at 30–70 °C. Optimal pH for *P. geniculata* AGEase (**b**), the reactions were performed in 50 mM sodium phosphate buffer (pH 5.0–9.0) at 60 °C. Thermostability of *P. geniculata* AGEase at 50 °C (**c**), the reactions were performed in 50 mM sodium phosphate buffer (pH 7.5). The pH stability of *P. geniculata* AGEase at pH 5.0–10.0 (**d**), the reactions were performed in 50 mM sodium phosphate buffer (pH 5.0–10.0) at 60 °C.

The crude enzyme was purified by gel column Superdex 200 chromatography. Purified product was subjected to SDS-PAGE and showed a single band at 43 kDa (Figure 1b). *P. geniculata* AGEase specific activity was 295.3 U·mg^−1^. The optimal temperature for *P. geniculata* AGEase was 60 °C (Figure 2a), and optimal pH was pH 7.5 (Figure 2b). Purified enzyme solution was incubated at 50 °C and sampled regularly to analyze residual enzyme activity to determine thermostability; the half-life of the enzyme at 50 °C was about 3 h (Figure 2c). Purified enzyme solution was incubated in 50 mM sodium phosphate buffer solution with different pH values at 4 °C for 24 h to determine pH stability. *P. geniculata* AGEase was stable at pH 7.0–8.5 (Figure 2d).

### 3.3. P. geniculata AGEase Substrate Specificity

In this study, d-glucose, d-fructose, d-mannose, d-lyxose, d-xylulose, d-xylose, *N*-acetyl-d-glucosamine, and *N*-acetyl-d-mannosamine were used as substrates for enzymatic conversion by *P. geniculata* AGEase at 50 °C and pH 7.5 (Table 2). *P. geniculate* AGEase could catalyze the isomerization between d-glucose and d-fructose; d-fructose and d-mannose; and d-lyxose and d-xylulose. The substrate specificity of *P. geniculata* AGEase was consistent with that of YihS reported by Itoh et al. [13].

**Figure 3:**
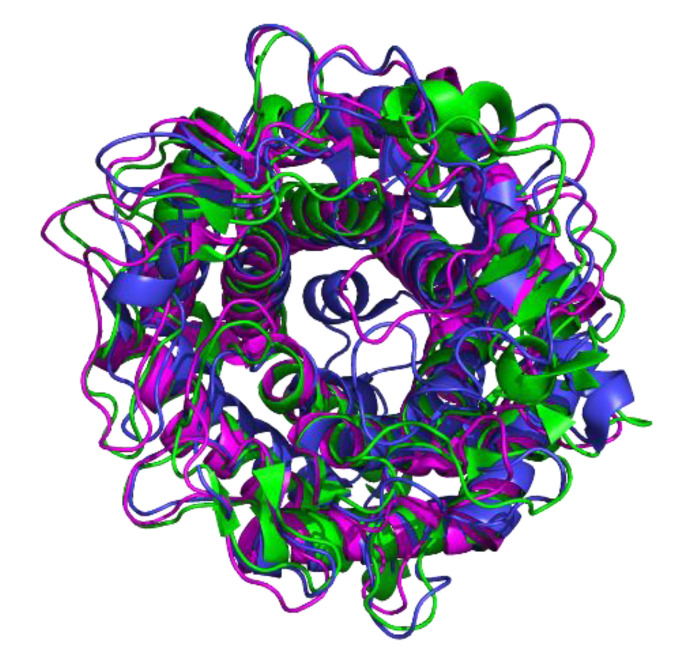
Three-dimensional structure of AGE enzyme family. *N*-acetyl-d-glucosamine 2-epimerase (PDB code: 1FP3) is highlighted in green; YihS enzyme (PDB code: 2AFA) is highlighted in blue; cellobiose 2-epimerase (PDB code: 3WKG) is highlighted in purple. The central region of this structure is the active site of the AGE family enzyme.

Although the amino acid sequence homology in the AGE family is low (20–40%), tertiary structures of family members have high homology, and all have an obvious (α/α)_6_-barrel motif (Figure 3) [11]. The tertiary structure study of the AGE enzyme family shows that the active centers of the *N*-acetyl-d-glucosamine 2-epimerase and YihS enzymes are significantly different. Since *N*-acetyl-d-glucosamine 2-epimerase catalyzes a modified sugar, the active center consists of two histidines, while the YihS enzyme active center is composed of three histidines. The three-dimensional structure of *P. geniculata* AGEase was simulated by using SWISS-MODEL. The three-dimensional structure of *P. geniculata* AGEase showed a catalytic center of three histidines: His179, His251, and His395, consistent with the active center of *Salmonella enterica* YihS enzyme (Figure S1) [13]. The results suggested that *P. geniculata* AGEase belonged to the YihS enzyme subfamily of the AGE enzyme family.

YihS enzyme is a kind of aldose-ketose isomerase that catalyzes interconversion between d-glucose and d-fructose; d-fructose and d-mannose; and d-lyxose and d-xylulose. To further determine the categorization of *P. geniculata* AGEase, the kinetics for different substrates were investigated (Table 3). *P. geniculata* AGEase had the lowest *K_m_* for d-mannose among these substrates, consistent with the reported substrate specificity of mannose isomerase [14]. Therefore, *P. geniculata* AGEase can be identified as d-MIase in the YihS enzyme of the AGE enzyme family.

### 3.4. Preparation of D-Mannose Using P. geniculata AGEase with d-Fructose as Substrate

**Figure 4:**
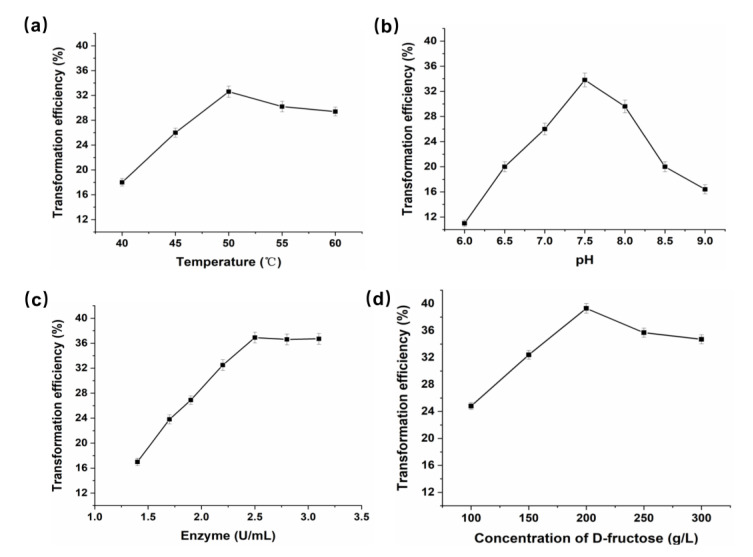
Effects of temperature, pH, enzyme concentration, and substrate concentration on D-mannose production. (**a**) Optimal temperature. The reactions were performed in 50 mM sodium phosphate buffer (pH 7.5) at 40–60 °C with 20% d-fructose and 2.5 U·mL^−1^ *P. geniculata* AGEase; (**b**) Optimal pH. The reactions were performed in 50 mM sodium phosphate buffer (pH 6.0–9.0) at 50 °C with 20% D-fructose and 2.5 U·mL^−1^ *P. geniculata* AGEase; (**c**) Optimal enzyme concentration. The reactions with different enzyme concentrations were performed in 50 mM sodium phosphate buffer (pH 7.5) at 50 °C with 20% D-fructose; (**d**) Optimal substrate concentration. The reactions with different substrate concentrations were performed in 50 mM sodium phosphate buffer (pH 7.5) at 50 °C with 2.5 U·mL^−1^ *P. geniculata* AGEase.

d-fructose was used as the substrate to generate d-mannose via catalysis by *P. geniculata* AGEase. The effect of temperature on the reaction was investigated at 40–60 °C (Figure 4a). The optimal temperature for generating d-mannose from d-fructose by *P. geniculata* AGEase was 50 °C. The effect of pH on the reaction was investigated at pH 6.0–9.0 (Figure 4b). The optimal pH of the reaction was pH 7.5. Between pH 6.0 and 7.5, conversion increased with pH increase; conversion decreased rapidly with increasing pH between pH 7.5 and 9.0. The impact of the amount of *P. geniculata* AGEase was investigated by adding different enzyme amounts at 50 °C and pH 7.5 with 250 g·L^−1^ of d-fructose as substrate. Increasing the amount of enzyme increased the conversion rate, with a highest rate of 36.9% within 2.5 U·mL^−1^ enzyme (Figure 4c). Further increasing the amount of enzyme did not increase the conversion rate.

The effect of d-fructose concentration on the reaction was investigated at optimal reaction conditions (50 °C, pH 7.5, and 2.5 U·mL^−1^ enzyme) with 100–300 g·L^−1^ d-fructose (Figure 4d). A total of 200 g·L^−1^ d-fructose yielded a maximum of 78.6 g·L^−1^ d-mannose after 2 h of reaction with a highest rate of 39.3%. The previously reported highest conversion rate of d-mannose from d-fructose catalyzed by *Pseudomonas* sp. No 2120 d-MIase was 36.7% with 200 g·L^−1^ substrate after a 32 h reaction [16]. The reaction time of *P. geniculata* AGEase was much shorter than that of *Pseudomonas* sp. No. 2120 d-MIase, which is suitable for d-mannose production.

## 4. Conclusions

The *age* gene encoding AGEase from *P. geniculata* was obtained and recombinant *P. geniculata* AGEase was successfully expressed in *E. coli* BL21 (DE3). *P. geniculata* AGEase was confirmed to be the d-MIase belonging to the YihS enzyme of the AGE enzyme family. *P. geniculata* AGEase exhibited a high conversion rate of 39.3% for d-mannose production from d-fructose. This identified *P. geniculata* AGEase has great value for d-mannose production.

## Figures and Tables

**Table 1:** Conversion efficiency of enzymes from different strains catalyzing substrate to d-mannose.

Source	Substrate	Conditions	Transformation Efficiency (%)
*Pseudomonas saccharophila*d-MIase	1.1 g/L d-fructose	30 °C, pH 7.4	29.0 [14]
*Agrobacterium radiobactor*d-MIase	250 g/L d-fructose	45 °C, pH 7.5, 2 h	29.2 [15]
*Pseudomonas* sp. d-MIase	200 g/L d-fructose	55 °C, pH 7.5, 32 h	36.7 [16]
*Caldanaerobius polysaccharolyticus*d-LIase	100 g/L d-fructose	65 °C, pH 6.5, 4 h	25.6 [17]
*Caldicellulosiruptor saccharolyticus* CEase	500 g/L d-glucose	75 °C, pH 7.5, 3 h	15.0 [18]

**Table 2:** Substrate specificity of recombinant *P. geniculata* AGEase.

Substrates	Products	Transformation Efficiency (%)
d-glucose	d-fructose	5.4
d-fructose	d-mannose	33.8
d-mannose	d-fructose	67.9
d-xylulose	d-lyxose	23.1
d-lyxose	d-xylulose	12.4
cellobiose	N.D.	N.D.
d-xylose	N.D.	N.D.
*N*-acetyl-d-mannosamine	N.D.	N.D.
*N*-acetyl-d-glucosamine	N.D.	N.D.

N.D. Not detected by assay conditions used.

**Table 3:** Kinetic parameters for recombinant *P. geniculata* AGEase.

Substrates	*k*_cat_ (s^−1^)	*K*_m_ (mM)	*k*_cat_/*K*_m_ (s^−1^·mM^−1^)
d-mannose	476.3 ± 4.0	49.2 ± 8.5	9.7 ± 0.5
d-fructose	176.5 ± 1.2	117.0 ± 22.5	1.5 ± 0.1
d-glucose	67.2 ± 0.9	382.0 ± 88.1	0.2 ± 0.01

## Supplementary Materials

The following are available online at https://www.mdpi.com/2304-8158/9/12/1809/s1, Figure S1: Active centers of AGE family epimerase/isomerase, Figure S2: High-performance liquid chromatography (HPLC) of d-fructose and d-mannose. The *age* gene encoding *P. geniculata* AGEase.
