# Supplementary material for: A Novel Pseudomonas geniculata AGE Family Epimerase/Isomerase and Its Application in d-Mannose Synthesis
Source: Foods. 2020 Dec 6;9(12):1809. doi: 10.3390/foods9121809 (PMC7762179; doi:10.3390/foods9121809)
Supplement: Supplementary file 1 [file foods-09-01809-s001.pdf]

A novel *Pseudomonas geniculata* AGE family epimerase/isomerase and its application in D-mannose synthesis

Zhanzhi Liu [a, b], Ying Li [a, b], Jing Wu [a, b], Sheng Chen [a, b*]

[a] State Key Laboratory of Food Science and Technology, Jiangnan University, 1800 Lihu Avenue, Wuxi 214122, Jiangsu Province, China

[b] School of Biotechnology and Key Laboratory of Industrial Biotechnology Ministry of Education, Jiangnan University,1800 Lihu Avenue, Wuxi 214122, Jiangsu Province, China

*Corresponding author:

chensheng@jiangnan.edu.cn

Figure Captions

Figure S1. Active centers of AGE family epimerase/isomerase. Catalytic residual amino acids of *Anabaena* sp. CH1 N-acetyl-D-glucosamine 2-epimerase: His239, His372 (a). Catalytic residual amino acids of *Salmonella enterica* YihS: His176, His258 and His386 (b). Catalytic residual amino acids of *P. geniculata* AGEase: His179, His251, His395 (c).

Figure S2. High-performance liquid chromatography (HPLC) of D-fructose and D-mannose. The retention times for D-fructose and D-mannose were 7.7 min and 8.8 min, respectively.

Figure S1.

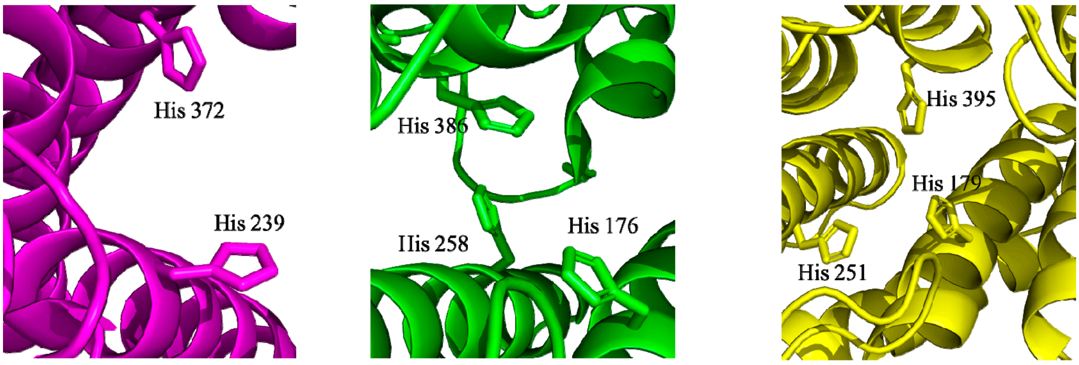

(A) **(B)** **(C)**

Figure S2.

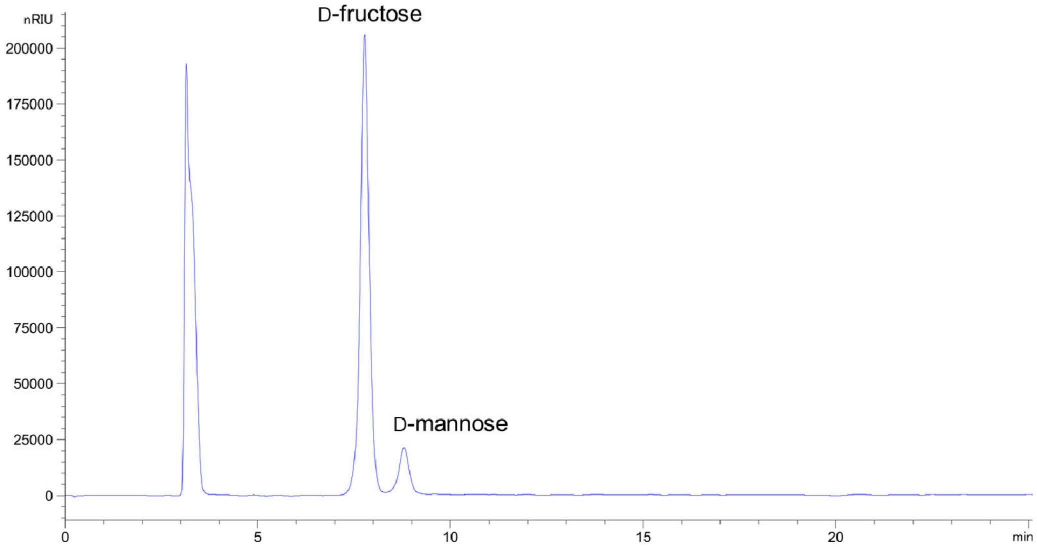
nRIU
200000
175000
150000
125000
100000
75000
50000
25000
0
D-fructose
D-mannose
0
5
10
15
20
min

The *age* gene encoding *P. geniculata* AGEase (RTY04456.1):

ATGAGCACCTCGCCCGATTTCCGTTCAGCCGCGTTCCTGCGTGCGCACATC
GCCGACACAATGGCGTTCTACCACCCGCGCTGCATCGATCCGAACGGCGG
CTTCTTCCACTACTTCCGTGACGACGGCAGCATCTACGATGCCAGCCACCG
CCACCTGGTGAGCAGCACCCGTTTCGTCTTCAACTACGCGATGGCCTACCG
CGAGTTCGGCAACGCCGAATACCGCGACGCGGTCGAGCACGGCGTGCGCT
ACCTGCGCGAGGTGCACCGCAACCCGGCCACCGGCGGCTACGCCTGGACC
CTGCGCGACGGCAAGGTCGAGGACGACATGAACCACTGCTATGGCGTGGC
GTTCGTGCTGCTGGCCTACAGCTGTGCGCTGAAGGCCGGCATCGAGCAGG
CGCGCGCCTGGATGGACGAGACCTGGCAACTGCTGGAAGCGCGCTTCTGG
GAGCCGCAGCACGGCCTGTACAAGGACGAGGCCGACGGCCAGTGGAACT
TCACCGGCTATCGCGGCCAGAACGCCAACATGCACATGTGCGAGGCGATG
CTGGCGGCGTTCGAGGCCAGTGGCGAACCGCGTTACGTCGAGCGTGCGCT
GCAGCTGGCCGACAACATGACCCGCCGCCAGGCCGCCAAGGCTGGCGGC
CTGGTCTGGGAGCACTACGATTCCAACTGGGAGATTGACTGGGACTACAA
CCTGGACGATCCCAAGCACCTGTTCCGCCCGTGGGGCTTCCAGCCGGGGC
ATCAGACCGAGTGGGCCAAGCTGCTGCTGATCCTCGATCGCCACGTGCAG
GCCGACTGGCTGGTGCCGACCGCGCAGCATCTGTTCGACGTGGCCGTGGC
GCGCAGCTGGGACGACGCGCGAGGCGGCCTGTACTACGGCTTCGCACCGG
AATCGCGCCGGCAGCCGGGCATGGAGGGCGCGCCGATCGGTGGCGACAG
CTTCGTCTGCGACGACGACAAGTACTTCTGGGTGCAGGCCGAAACGCTGG
CCACCGCCGCACTGATGGCCAAGCGCACCGGTGATGACCGCTACTGGCAG
TGGTACGAGCGCATCTGGGCGTACGCGTGGGAGCACTTCGTCGACCATCA
GTACGGCGCCTGGTTCCGCATCCTCGATGCCGACAACCGCAAGTACAGCG
ACGAGAAGAGCCCGGCCGGCAAGGTGGATTACCACACCATGGGCGCGTG
CTACGAAGTGTTGAACGTGGTGCGTTGA
